# Antibiotic Prophylaxis in Reduction Mammaplasty: A Network Meta-Analysis

**DOI:** 10.1007/s00266-023-03313-2

**Published:** 2023-03-16

**Authors:** Konstantinos Seretis, Nikolaos Bounas, Foteini Papaspyrou

**Affiliations:** Department of Plastic Surgery, Medical School, University of Ioannina, Leoforos St. Niarchou, 45500 Ioannina, Greece

**Keywords:** Reduction mammaplasty, Antibiotics, Breast, Infection, Wound healing, Breast reduction

## Abstract

**Background:**

Mounting evidence suggests that breast reduction surgery displays higher rates of surgical site infections (SSI) than initially presumed. Objective of this network meta-analysis is to evaluate the effectiveness of different antibiotic regimens in the prophylaxis from surgical site infections and delayed wound healing (DWH) following breast reduction.

**Methods:**

A network meta-analysis was conducted using a predetermined protocol after searching the electronic databases MEDLINE, Scopus, the Cochrane Library and US National Institutes of Health Ongoing Trials Register from inception to July 2022. The included studies had to examine breast reduction in females with at least 1-month follow-up, receiving antibiotics in an intervention arm compared to a control arm. The quality of studies was assessed using the Cochrane risk of bias tool. A frequentist Mantel-Haenszel approach was adopted for the reported SSI rates while an inverse variance random effects model was used for the DWH rates.

**Results:**

A total of 10 studies was included in the analysis involving 1331 patients. All but one study controlled for major risk factors, and no differences were observed in patients’ baseline characteristics. Antibiotic administration significantly reduced the SSI rate after breast reduction, with the prolonged antibiotic regimen being the most efficacious (odds ratio [OR]: 0.36 [95%CI: 0.15–0.85]). No statistically significant reduction in delayed wound healing rate was revealed among the regimens.

**Conclusions:**

Antibiotics mitigate the SSI rate after breast reduction. This meta-analysis provides an evidence-based strategy to optimize antibiotic administration. Further research is needed though to examine antibiotic prophylaxis on delayed wound healing.

**Level of Evidence III:**

This journal requires that authors assign a level of evidence to each article. For a full description of these Evidence-Based Medicine ratings, please refer to the Table of Contents or the online Instructions to Authors www.springer.com/00266.

**Supplementary Information:**

The online version contains supplementary material available at 10.1007/s00266-023-03313-2.

## Key points

1. This study highlights the beneficial effect of antibiotics in the reduction of surgical site infection (SSI) rates following reduction mammaplasty.
2. A prolonged postoperative antibiotic regimen is associated with the least of SSI rates.
3. Antibiotic administration does not affect significantly the delayed wound healing rates after reduction mammaplasty.
4. The rigorous methodology of this study design and the exceptional matching of the patients’ baseline characteristics allow high confidence in the reported outcomes.

**Supplementary Information:**

The online version contains supplementary material available at 10.1007/s00266-023-03313-2.

## Introduction

Breast reduction (BR), also known as reduction mammaplasty, presents one of the most popular aesthetic operations performed worldwide [1]. Interestingly, 82,643 operations were performed in the USA alone in 2021, marking a 49% annual increase in the post-pandemic era [1]. This fact is principally attributed to the constantly improved outcomes and persistent effort for low complication rates.

BR has historically been defined as a ‘clean’ procedure due to presumably low surgical-site infection (SSI) rates, ranging between 1% and 2%, thus antibiotic prophylaxis is not recommended according to the CDC’s 1999 Guideline for Prevention of Surgical Site Infection [2]. However, there have been numerous studies throughout the years that suggested much higher SSI rates, in the range of 4% and 36% [3]. These rates, which nominally correspond to either a ‘clean-contaminated’ or ‘contaminated’ procedure, support the antibiotics’ administration to prevent SSIs, which can lead to a multitude of adverse outcomes, including delayed wound healing, prolonged hospital stay, and increased costs [4, 5]. A recent meta-analysis, focused on SSI rates in breast surgery, supports the notion that breast surgery should be removed from the ‘clean’ classification, while also concluding that antibiotics reduce the frequency of SSIs [6]. In BR specifically, the relevant systematic reviews provide conflicting evidence regarding antibiotic administration for prevention of SSIs [3, 7, 8].

The aim of this network meta-analysis is to combine the evidence from different antibiotic regimens and evaluate their effectiveness in the prophylaxis from SSI following breast reduction in order to identify the optimal strategy.

## Materials and Methods

A network meta-analysis was conducted using a predetermined protocol established according to the Cochrane Handbook’s recommendations [9]. The review adhered to the updated PRISMA (Preferred Reporting Items for Systematic Reviews and Meta-Analyses) guidelines (Supplemental Digital Content S1 Table) [10]. The review protocol was registered at PROSPERO (registration no. CRD42022350724).

### Search Strategy

An electronic literature search in MEDLINE (PubMed), Scopus, the Cochrane Library and US National Institutes of Health Ongoing Trials Register electronic databases was conducted from inception to July 2022. The string search [“*breast reduction*”] and [*“antibiotic”*] was applied*.* No time and language restriction were applied. This search was supplemented by a review of reference lists of potentially eligible studies and a manual search of key journals in the field of plastic surgery.

### Eligibility of Relevant Studies

**Fig. 1:**
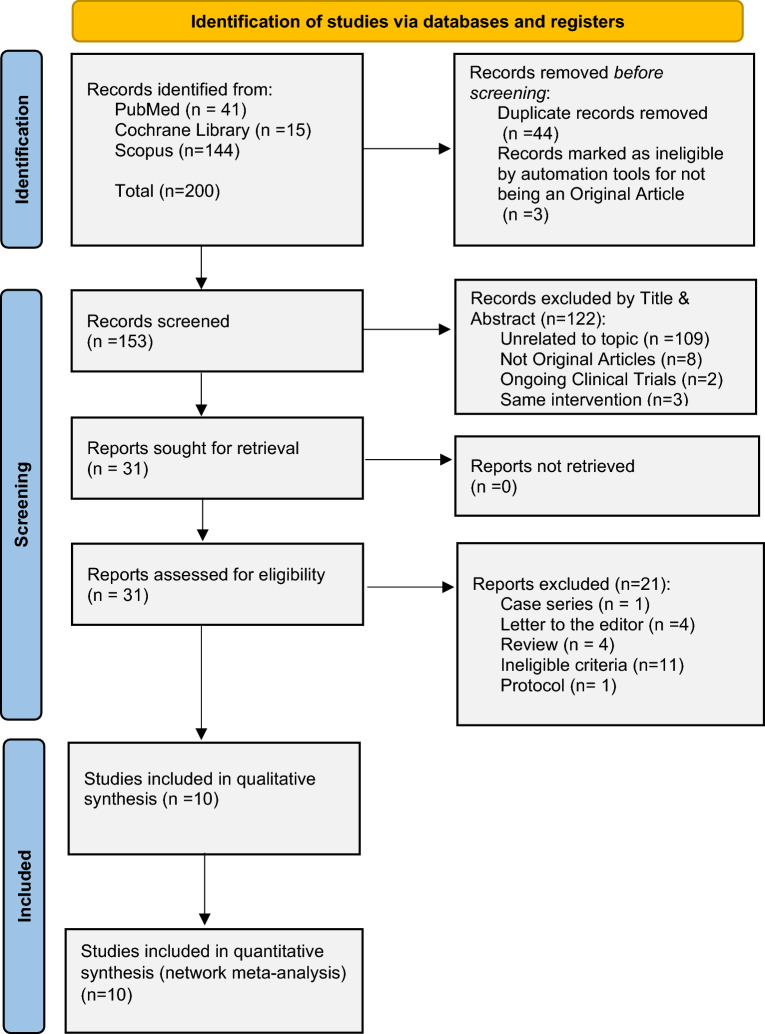
Meta-analysis flowchart

To be included in this review, studies had to examine breast reduction in females with at least 1-month follow-up, receiving antibiotics in an intervention arm compared to a control arm. The intervention groups included administration of antibiotics only preoperatively (preop group/PrG), preoperatively and postoperatively for less than 2 days (short postop group/SPoG) or more than 4 days (long postop group/LPoG). The included studies reported data on postoperative infection rates, wound healing problems; and were published in a peer-reviewed journal. We excluded studies with less than 10 subjects, studies reporting only on surgical technique, and review articles, duplicate reports, editorials and correspondences (Fig. 1). There were no restrictions on the antibiotic or dose administered.

### Study Selection

Two reviewers (K.S. and N.B.) independently screened retrieved database files and the full text of potentially eligible studies for relevance. Disagreement was resolved by consensus.

### Data Collection and Risk of Bias Assessment

Data extraction was conducted independently by 2 authors (K.S. and N.B.) using a standardized form. Discrepancies were resolved by consensus. The reviewers extracted data, including the general study characteristics, population characteristics, and outcomes of interest. Primary outcome was the incidence of SSI, and secondary outcome was the delayed wound healing (DWH) rate.

The quality of studies was assessed using the Cochrane risk of bias tool.

### Data Synthesis and Analysis

Network meta-analysis was conducted to collectively compare different antibiotic regimens. All interventions of the included studies were constructed as a network map, and appropriate network graphs were constructed to facilitate their visualization. The control group was designated as the reference group. Relative treatment effects across studies were expressed in terms of odds ratios (ORs) and associated with 95% confidence intervals (CIs). For each outcome of interest, heterogeneity and inconsistency between studies was assessed using I^2^, the generalized DerSimonian-Laird estimate of tau^2^, and Cochran’s Q statistic. We fitted a frequentist Mantel-Haenszel fixed effect model for SSI rate, owing to the decreased heterogeneity observed across the included studies (Q=1.91, p=0.75), while the inverse variance model was utilized to assess DWH rate, as significant heterogeneity was observed [I^2^=72.1%, (Q=10.74, p=0.01)] [11]. The transitivity assumption underlying the NMA was evaluated by checking the distribution of clinical and methodological variables that could potentially act as effect modifiers across treatment comparisons (such as the study design/approach, and baseline measures of the relevant variable).

The pairwise comparisons of interventions were shown in a league table and a forest plot. The probability of the most efficient intervention was estimated and ranked using the P-Score, which measures the certainty that one treatment is better than the other, averaged over all competing treatments, and the closer the P-Score is to 1, the better the therapeutic effect of the intervention may be [12]. The confidence, and hence quality of evidence, for each outcome was rated according to the grading of recommendations assessment, development, and evaluation (GRADE) system with the support of the CINeMA software (Confidence in Network Meta-Analysis) [13, 14]. Publication bias was examined by the assessment of comparison-adjusted funnel plots. This network meta-analysis was conducted using the “netmeta” package in R (version 4.2.1, R Foundation for Statistical Computing, Vienna, Austria).

## Results

The study selection process is summarized in Figure 1. From a total of 200 records, 10 studies met the inclusion criteria and were included in the data analysis (Table 1) [15–24].

**Table 1:** Synopsis of the network meta-analysis

Outcomes	No. of studies	No. of patients	No. of pairwise comparisons	No. of designs	I^2^	Consistency test		Publication bias
						Global *P* value	Local *P* value	
Surgical site infections	10	1331	12	6	–	0,91	All insignificant	Egger’s Test insignificant (p=0.55)
Delayed wound healing	5	537	7	5	71%	0,02	All insignificant	Funnel plot symmetry observed

The 10 studies included were conducted in the USA (3), Brazil (2), Canada (2), Sweden (1), Switzerland (1), and Norway (1). All studies but one conducted in a single institution and were published between 1990 and 2022. There were 4 randomized controlled trials (RCT), 2 prospective (PCS), and 4 retrospective controlled studies (RCS), involving a total of 1331 patients (Table 2). The antibiotic regimens used for SSI prevention following reduction mammaplasty are shown in Table 2. Prophylactic antibiotics were administered either as a single dose preoperatively (PrG) or continued postoperatively for 24-48 hours (SPoG) or 4-7 days (LpoG). Overall, 6 PrG, 5 LpoG, and 3 SpoG studies were identified.

**Table 2:** Studies involving reduction mammaplasty that successfully passed both levels of screening and were included in the meta-analysis

Author, Year	Study period	Study design	Intervention group	Antibiotic	*N *(patients)	Control group	*N *(Patients)
Platt et al., 1990	1985–1987	RCT	Preop	Cefonicid	18	Placebo	15
Serletti et al., 1994	1985–1989	RCS	Preop + postop 1-2d	1st gen. Cephalosporin	47	No ABs	59
Amland et al., 1995	1991–1992	RCT	Preop	Azithromycin	25	Placebo	22
Kompatscher et al., 2003	1997–2001	RCS	Preop	Cefuroxime	153	No ABs	136
Ahmadi et al., 2005	2000–2001	PCS	Preop Preop + Postop 4d	Cefazolin/ Cephalexin	17 17	No ABs	16
O’Grady et al., 2005	2000–2002	RCS	Preop + Postop 1d Preop + Postop 6d	Cephalexin	80 53	NSG	–
Veiga-Fihlo et al., 2010	2006–2009	PCS	Preop + Postop 6d	Cephalothin/ Cephalexin	50	No ABs	50
Lewin et al., 2015	2009–2013	RCT	Preop	Cloxacillin	162	No ABs	163
Garcia et al., 2020	2019–2020	RCT	Preop + Postop 7d Preop + Postop 1d	Cephalothin/ Ciprofloxacin	62 62	NSG	–
Doucet et al., 2022	2018–2019	RCS	Preop Preop + Postop 6d	Cefazolin/ Cefalexin	62 62	NSG	–
Total					870		461

*RCT* Randomized controlled trial, *RCS* Retrospective controlled study, *PCS* Prospective controlled study, *ABs* Antibiotics, NSG: No study group

The risk of bias was considered low for the 4 RCTs and intermediate for the 2 prospective and 4 retrospective studies. Publication bias was assessed by visual inspection of the funnel plots and Egger’s statistical test if applicable (Figures, Supplemental Digital Content 2,3). A relative symmetry was consistently observed. The results of the netsplit function evaluating local inconsistency are described in the provided forest plots (Figures, Supplemental Digital Content 4, 5). The quality of evidence was rated according to the GRADE system (Table 1), and the confidence rating of each comparison using CINeMA is described in the supplement file (Figures, Supplemental Digital Content 6,7).

The meta-analysis included 1331 breast reduction patients; 870 in the intervention and 461 in the control group. The intervention groups included 437 patients in the preoperative only group, 189 and 244 patients in the short and long postoperative groups, respectively. Individuals’ baseline characteristics are presented in Table 3. Similar characteristics were revealed among the groups, in terms of age, BMI, smoking and other well-known risk factors, breast weight resected, and duration of operation. Consequently, the groups were well-matched at baseline along variables relevant to risk factors for SSI following breast reduction, and thus data were integrated to make direct comparisons between intervention and control groups.

**Table 3:** Baseline characteristics of the study groups included in the meta-analysis

Study	Group	Age	BMI	Smoking	Weight resected	Duration (Time)	Other risk factors controlled
Platt et al., 1990	I C	51.6 53.5	25.6± 5 24.8± 4.5	NR	NR	NR	Diabetes, Hospitalization, previous breast surgery
Serletti et al., 1994	I C	38 32	NS	23% 15%	721 719	NR	Diabetes
Amland et al., 1995	I C	NS	NR	NR	NR	NR	–
Kompatscher et al., 2003	I C	34±13 33±13	24± 3.1 25± 3.5	0 0	385* 450	190* 160	Diabetes, ASA
Ahmadi et al., 2005	I I C	34 36 32	33 33 31	0 5.9% 17.6%	794 1235 962	204 235 215	Diabetes, Steroid use, PVD, previous breast surgery
O’Grady et al., 2005	I C	NS	NR	NS	NR	NR	–
Veiga-Fihlo et al., 2010	I C	36.3± 5.4 33.3±10.4	24.8±1.8 24.5±1.8	NR	755 931	193±29 202±34.9	–
Lewin et al., 2015	I C	39.5±15.5 40.3±15.8	24.5±2.3 24.5±2.1	13% 9.8%	488 466	115±39.5 112±40.4	Diabetes, ASA, Drainage, Bleeding volume
Garcia et al., 2020	I I	29 34.5	25 24	NR	695 790	180* 200	–
Doucet et al., 2022	I C	48.1* 40.3	33* 30	16.1% 9.7%	1196 1109	108 113	Diabetes, Drainage

*I* Intervention, *C* Control, *NS* Not significant, *NR* Not reported

*p<0.05

**Fig. 2:**
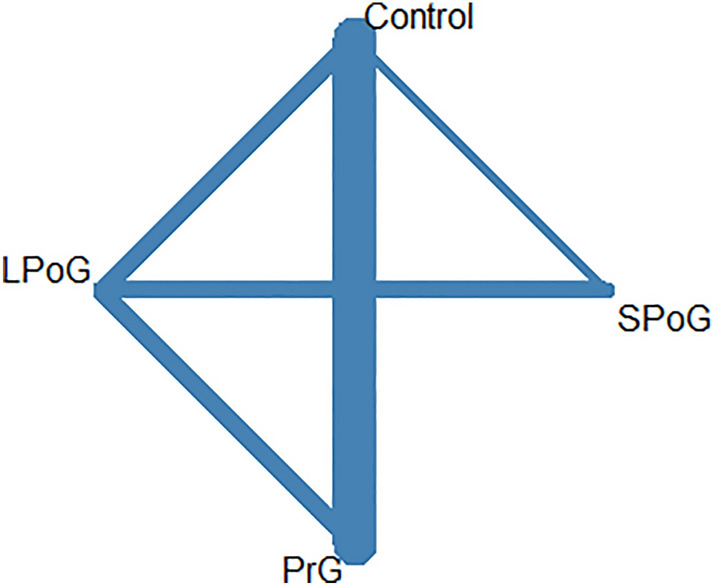
The network map for surgical site infection among different antibiotic regimens

**Fig. 3:**
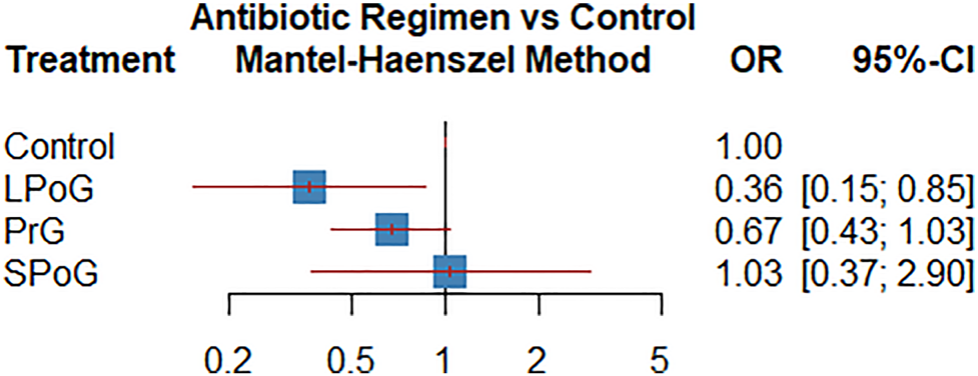
Forest plot for surgical site infection among different antibiotic regimens

The network map for surgical site infection among different antibiotic regimens is shown in Fig. 2. The pairwise comparisons are shown in the league table (Table 4A) and the forest plot, providing the summary of the results (Fig. 3). Based on P-Score, the long postoperative antibiotic group was the regimen most likely to show the lowest rate of infection (0.96), followed by preoperative only group (0.61), SPoG (0.23), and control group (0.19) (Table 5A).

**Table 4:** League tables of comparisons between antibiotic regimens in terms of A. Surgical site infections B. Delayed wound healing

A.			
**Control**	3.06 (0.95–9.87)	1.50 (0.96–2.35)	0.78 (0.18–3.31)
2.77 (1.17–6.53)	**LPoG**	0.54 (0.18–1.64)	0.40 (0.13–1.20)
1.50 (0.97–2.33)	0.54 (0.23–1.30)	**PrG**	NA
0.97 (0.34–2.71)	0.35 (0.14–0.89)	0.64 (0.22–1.88)	**SPoG**
B.			
**Control**	6.22 (0.38–101.16)	22.75 (1.32–393.48)	1.39 (0.14–14.16)
5.33 (0.74–38.15)	**LPoG**	1.09 (0.19–6.24)	0.66 (0.10–4.18)
6.77 (0.70–65.77)	1.27 (0.23–7.00)	**PrG**	NA
2.45 (0.37–16.09)	0.46 (0.09–2.36)	0.36 (0.04–3.35)	**SPoG**

OR (95% CI)

**Table 5:** Ranking of antibiotic regimens in terms of A. Surgical site infections B. Delayed wound healing, based on their P-Score

Antibiotic regimen	P-Score
*A.*	
LPoG	0.9637
PrG	0.6138
SPoG	0.2325
Control	0.1900
*B.*	
PrG	0.7913
LPoG	0.7226
SPoG	0.3950
Control	0.0911

**Fig. 4:**
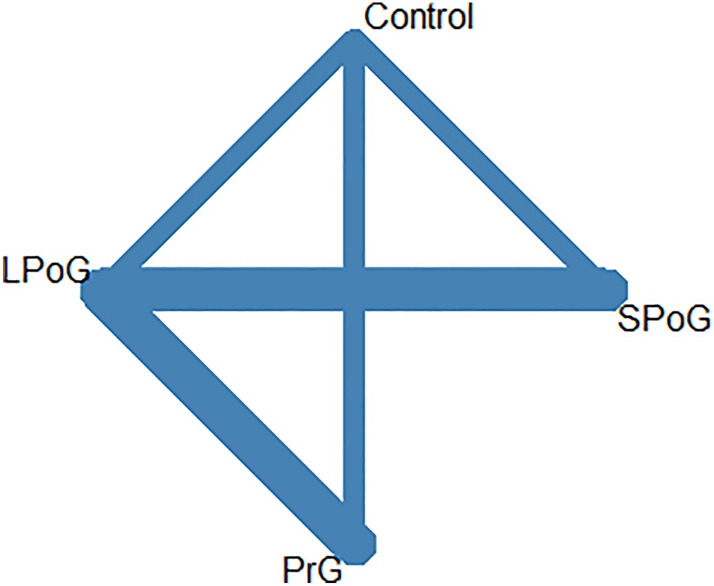
The network map for delayed wound healing among different antibiotic regimens

**Fig. 5:**
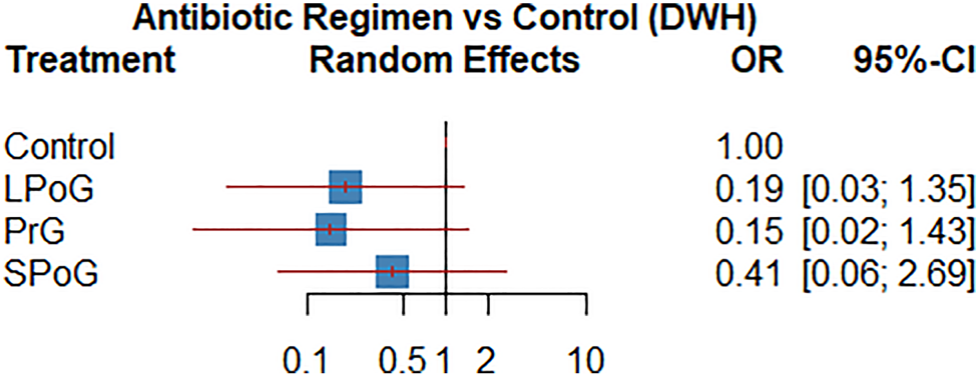
Forest plot for delayed wound healing among different antibiotic regimens

The network map for wound healing problems is shown in Fig. 4 and the pairwise comparisons were evaluated in the league table (Table 4B) and the forest plot (Fig. 5). Based on P-Score, PrG ranked best for wound healing (0.79), followed by LPoG (0.72), SPoG (0.4) and control group (0.09) (Table 5B).

## Discussion

Although there is evidence regarding antibiotics use following reduction mammaplasty, previous studies have not been able to demonstrate a relative superiority of any of the different regimens due to insufficient synthesis of the evidence. Network meta-analysis is a technique for comparing three or more interventions simultaneously in a single analysis by combining both direct and indirect evidence across a network of studies, allowing estimation of the ranking and hierarchy of interventions, if performed correctly [11, 25]. In this network meta-analysis, we summarized the best available data on the impact of antibiotic prophylaxis following breast reduction on SSI and delayed wound healing, and showed a prophylactic effect on SSI rate, with the lower incidence identified after prolonged antibiotic administration, whereas wound healing was not affected by antibiotics significantly.

These findings confirm the relevant evidence from previous studies, but also question the common practice of short (24 hours) perioperative antibiotic administration. The long course, and the preoperative only administration, though not significant, showed the best ranking in this network meta-analysis. In the era of evidence-based medicine and individualized patient treatment, the findings of this study provide evidence on optimal strategy, focusing also on the patient characteristics. Although the SSI risk following breast reduction is higher than other ‘clean’ procedures, the decision, regarding prolonged or preoperative only administration of antibiotics, may be based on the individual patient risk factors. Hardwicke et al. conducted a systematic review in 2013, comprising data from 7 studies, which claimed that a single dose of systemic perioperative antibiotic prophylaxis can significantly reduce the SSI rates following breast reduction [7]. Similarly, the meta-analysis of Shortt et al. demonstrated a 75% reduction in wound infections with preoperative antibiotics [3]. However, no conclusions could be made concerning the effect of postoperative antibiotics on SSI rates following breast reduction due to insufficient data. The meta- analysis of Zapata-Copete et al. in 2017, included two more relevant studies with 470 more patients compared to Shortt et al., but excluded studies with extended postoperative antibiotic administration [8]. They found lower incidence of SSIs when antibiotic prophylaxis was administered [8]. The prophylaxis group had 8% less risk of SSI, while the number needed to treat (NNT) was estimated as 12 patients.

Interestingly, in a large community-based retrospective study of 855 patients undergoing elective, non-reconstructive clean breast operations (including breast reduction), the prophylactic administration of a single antibiotic dose did not reduce the SSI rate [26]. They concluded that BMI >25 kg/m^2^ and the use of an inadequate prophylactic dose of antibiotics may increase the risk of SSI. Considering that obesity is a risk factor for SSI and wound healing problems, this NMA included comparative studies with normal weight, overweight and obese patients’ groups, which were well-matched at baseline. Guidelines from the Surgical Care Improvement Project state that prophylactic antibiotics should be administered within 1 hour before surgical incision, targeted against the most probable bacteria, and discontinued within 24 hours after the surgery [27]. The recent revised clinical practice guidelines of the American Society of Plastic Surgeons regarding breast reduction are based on these guidelines, and thus suggest against the routine use of postoperative oral antibiotics [28].

This review supports this thesis but also provides evidence of a positive effect on SSI rates from a prolonged antibiotic administration. A recent Brazilian national survey showed that the vast majority (95%) of the respondents administer at least a single prophylactic dose, and the 75% maintain antibiotics for additional days after discharge [29]. This group of surgeons reported significantly lower SSI rates compared to the surgeons administering antibiotics only preoperatively (1.4% compared to 25.9% had a 5–10% SSI rate, respectively). These outcomes should be interpreted with caution due to the low response rate and the inherited survey limitations. We should also not forget that widespread antibiotic use is associated with the risk of allergic reaction, antibiotic-specific side effects, natural flora suppression, secondary infections, bacterial resistance, and increased costs [19]. Garcia et al. conducted a randomized control trial, implementing Surgical Care Improvement Project guidelines, in order to compare 1-day to 7-day antibiotic administration after reduction mammoplasty [23]. The study reported a very low SSI rate (0.8%) and found no statistical differences in infection or other surgical complications rates, suggesting that antibiotics should be discontinued within 24 hours after surgery. Although the sample size was calculated, based on data from a previous nonrandomized study, the sample size may have been insufficient to reject the null hypothesis that is actually false (type II statistical error), due to the low SSI rate. This problem can be solved by a multicenter clinical trial or even better by a well-designed and executed meta-analysis, and thus we attempted to conduct one.

Among the strengths of this study is the rigorous methodology used, including the best available evidence to answer a controversial question. The different groups studied had similar baseline characteristics, thus limiting the potential bias of different study groups and designs to the primary outcome of interest. This fact was also expressed by the decreased heterogeneity identified, regarding SSI rate outcome. In addition, no publication bias was revealed, further enhancing the outcomes of the meta-analysis. On the contrary, the secondary outcome of DWH rate was estimated by a rather small number of studies, and thus this outcome may be further evaluated in well-designed clinical trials in the future.

## Conclusions

Overall, antibiotic prophylaxis has proven to be efficacious in the mitigation of SSI rate after breast reduction. The outcomes of this study suggest that prolonged postoperative antibiotic administration lowers the SSI incidence more than the other antibiotic regimens. The antibiotics’ role, however, in the process of wound healing is not significant. Undeniably, more research will shed light on the different antibiotic regimens and cement their true effect size.

## Supplementary Information

Below is the link to the electronic supplementary material.

Supplementary file 1 Prisma checklist.

Supplementary file 2 Funnel plot for SSI.

Supplementary file 3 Funnel plot for DWH.

Supplementary file 4 Netsplit forest plot for SSI.

Supplementary file 5 Netsplit forest plot for DWH.

Supplementary file 6 Confidence rating for SSI using CINeMA.

Supplementary file 7 Confidence rating for DWH using CINeMA.
