# Supplementary figures and images for: Antibiotic Prophylaxis in Reduction Mammaplasty: A Network Meta-Analysis
Source: Aesthetic Plast Surg. 2023 Mar 16;47(3):1009–17. doi: 10.1007/s00266-023-03313-2 (PMC10229481; doi:10.1007/s00266-023-03313-2)

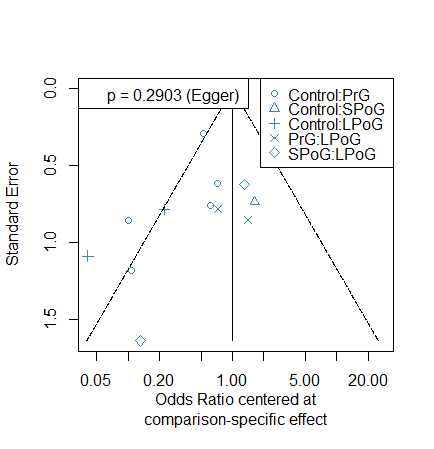

Supplement: Supplementary file 2 — Funnel plot for SSI. [file 266_2023_3313_MOESM2_ESM.png]

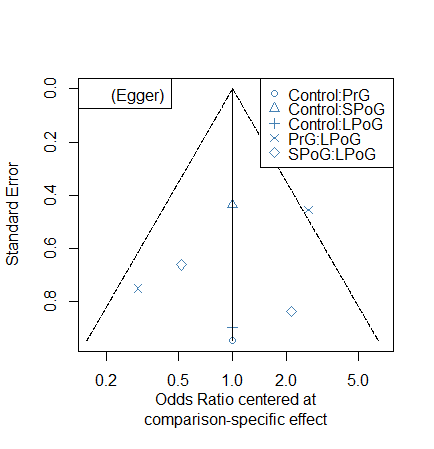

Supplement: Supplementary file 3 — Funnel plot for DWH. [file 266_2023_3313_MOESM3_ESM.png]

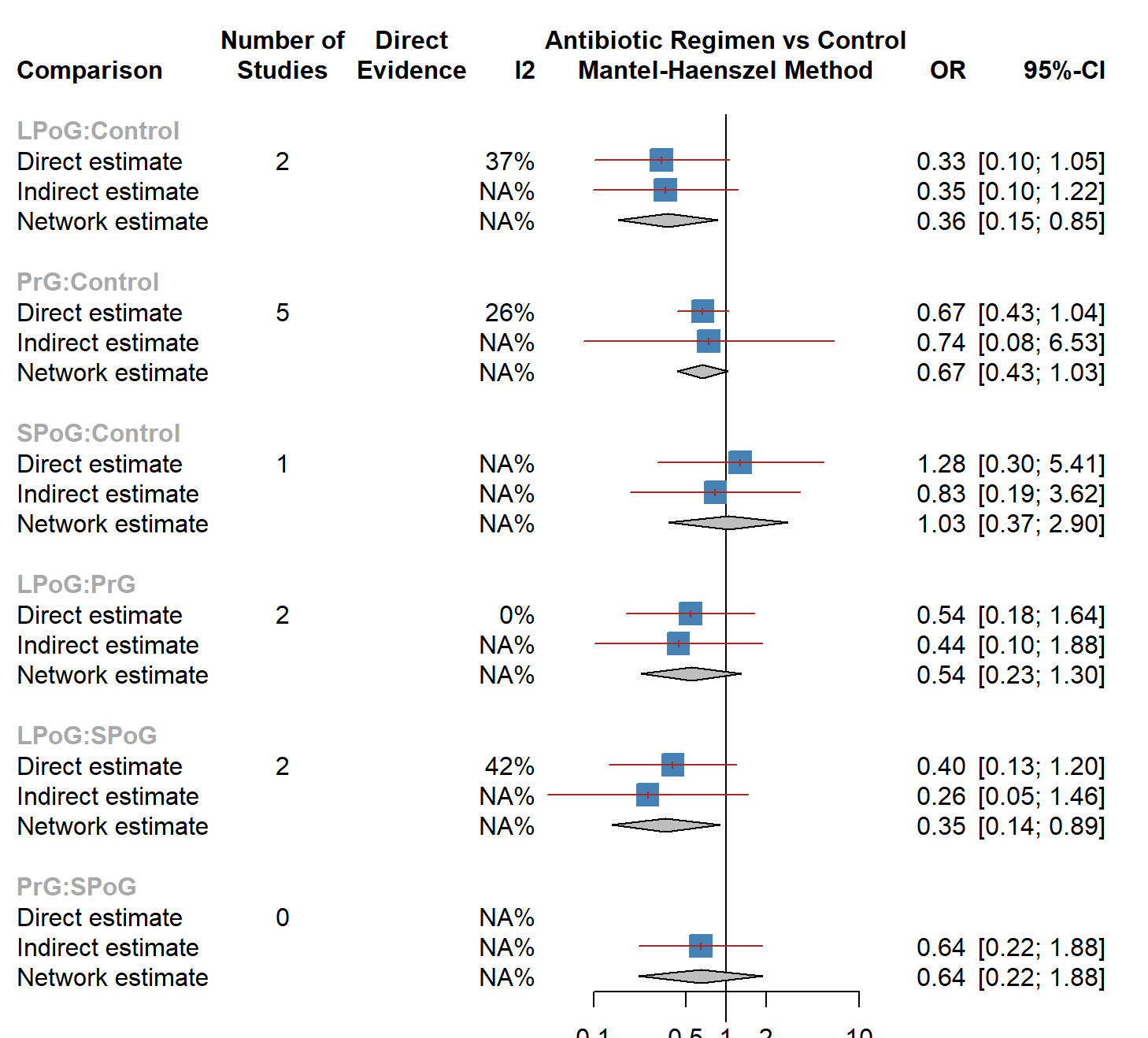

Supplement: Supplementary file 4 — Netsplit forest plot for SSI. [file 266_2023_3313_MOESM4_ESM.png]

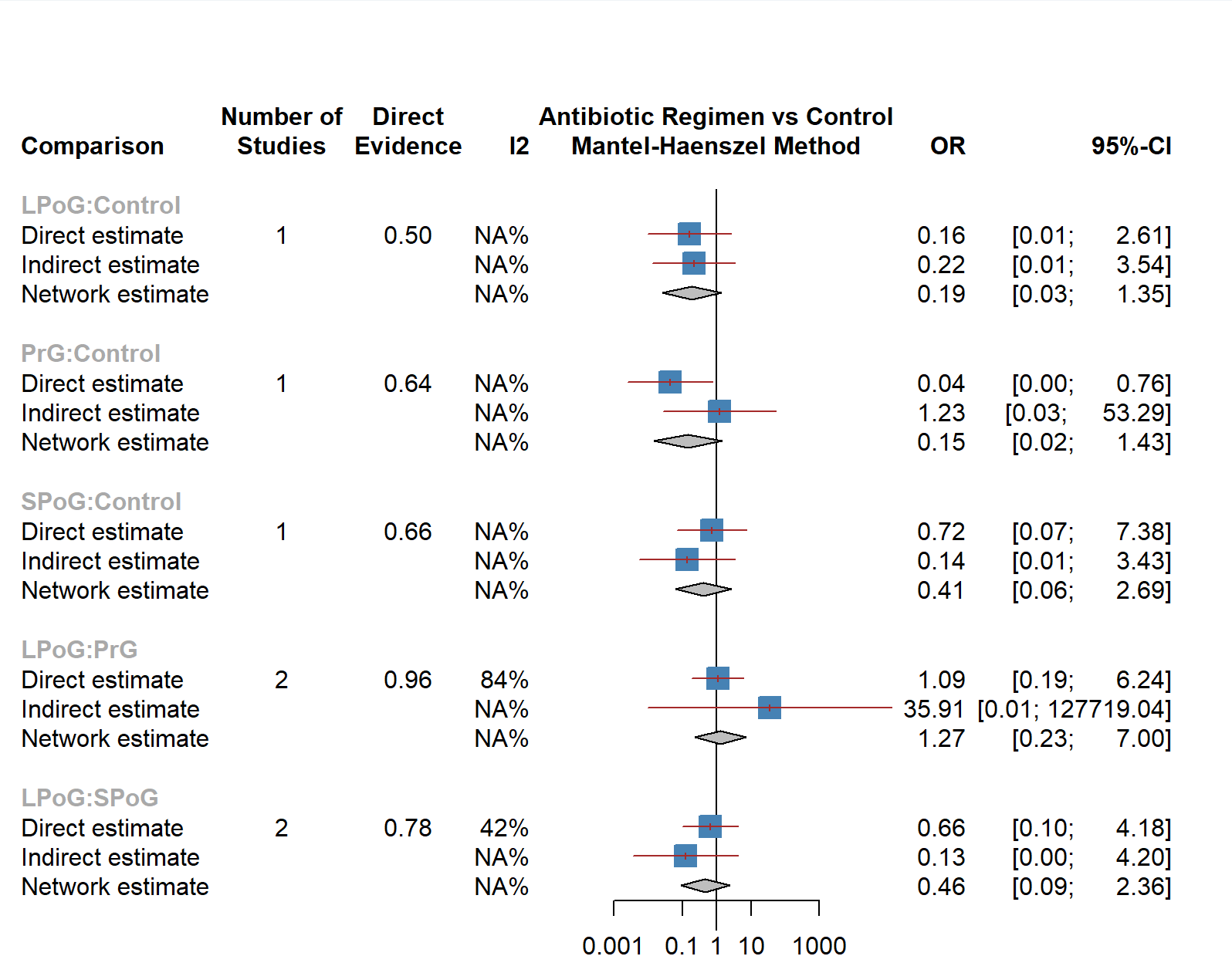

Supplement: Supplementary file 5 — Netsplit forest plot for DWH. [file 266_2023_3313_MOESM5_ESM.png]

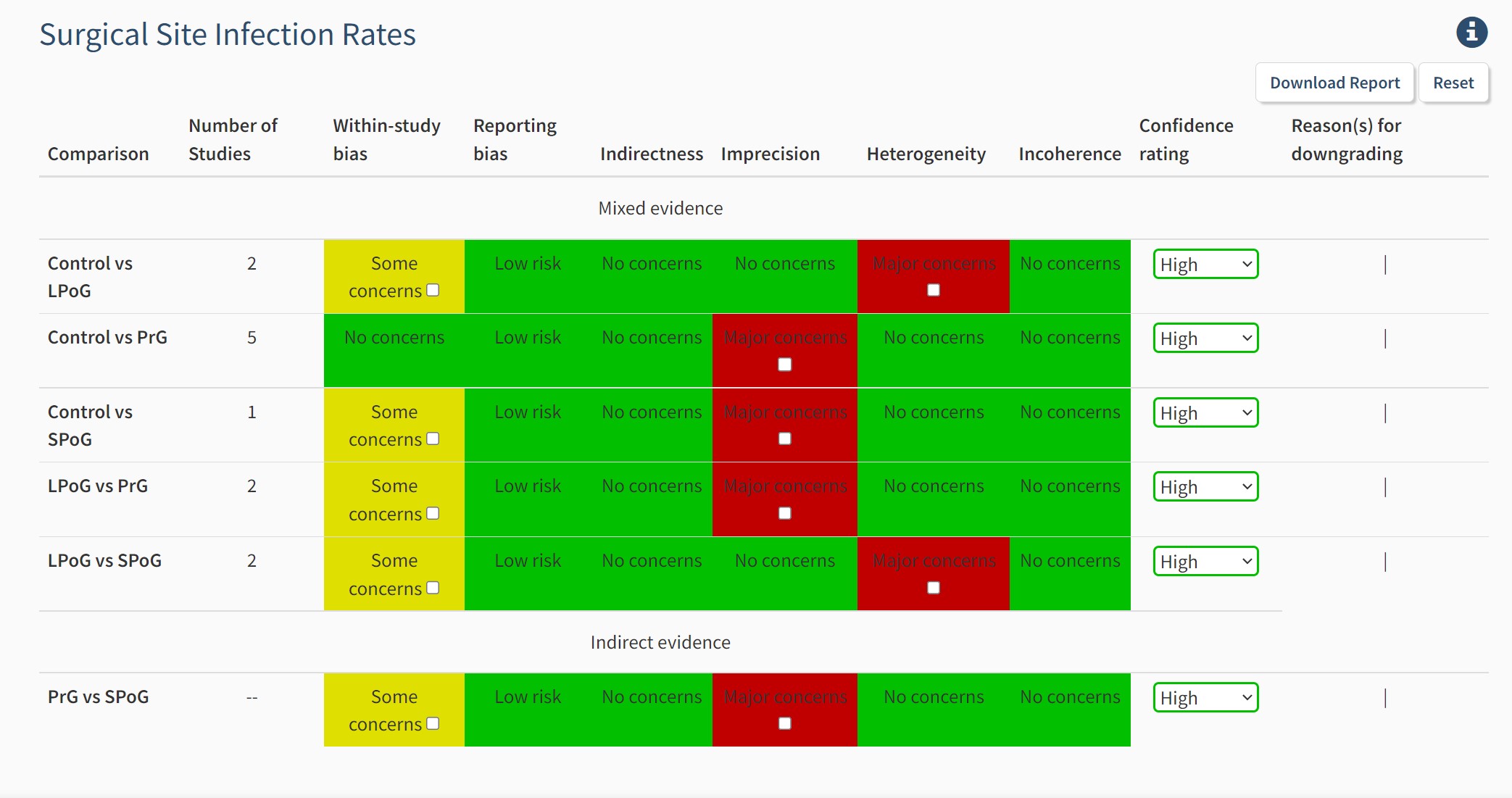

Supplement: Supplementary file 6 — Confidence rating for SSI using CINeMA. [file 266_2023_3313_MOESM6_ESM.jpg]

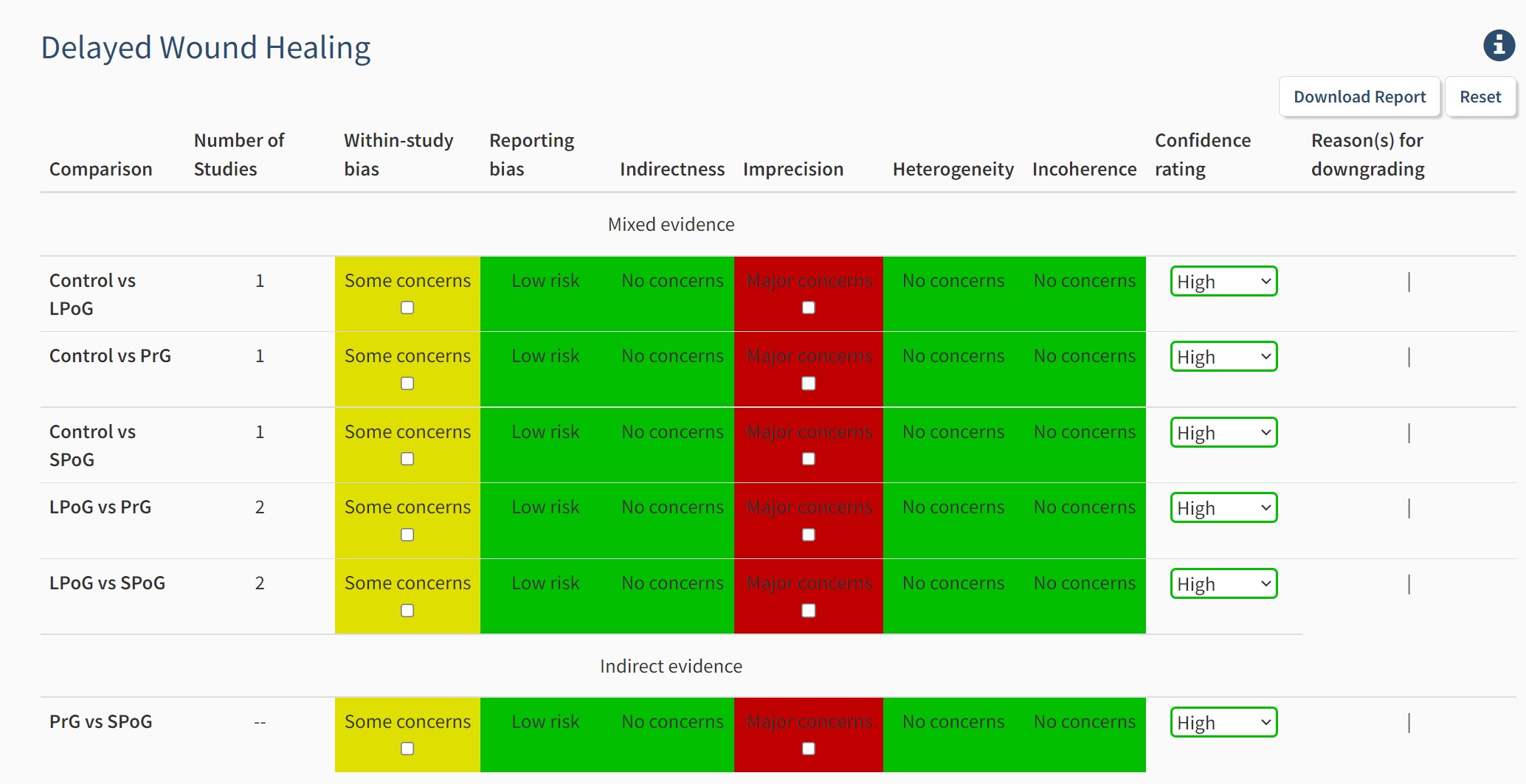

Supplement: Supplementary file 7 — Confidence rating for DWH using CINeMA. [file 266_2023_3313_MOESM7_ESM.jpg]
